# Therapeutic Approach to Low-Grade Serous Ovarian Carcinoma: State of Art and Perspectives of Clinical Research

**DOI:** 10.3390/cancers12051336

**Published:** 2020-05-23

**Authors:** Angiolo Gadducci, Stefania Cosio

**Affiliations:** Department of Clinical and Experimental Medicine, Division of Gynecology and Obstetrics, University of Pisa, 56127 Pisa, Italy; stefania.cosio@gmail.com

**Keywords:** low grade ovarian serous carcinoma, mitogen activated protein kinase (MAPK), surgery, chemotherapy, endocrine therapy, molecularly-targeted agents

## Abstract

Low-grade serous ovarian carcinoma (LGSOC) is a distinct pathologic and clinical entity, characterized by less aggressive biological behavior, lower sensitivity to chemotherapy and longer survival compared with high-grade serous ovarian carcinoma. LGSOC often harbors activating mutations of genes involved in mitogen activated protein kinase (MAPK) pathway. Patients with disease confined to the gonad(s) should undergo bilateral salpingo-oophorectomy, total hysterectomy and comprehensive surgical staging, although fertility-sparing surgery can be considered in selected cases. Women with stage IA-IB disease should undergo observation alone after surgery, whereas observation, chemotherapy or endocrine therapy are all possible options for those with stage IC-IIA disease. Patients with advanced disease should undergo primary debulking surgery with the aim of removing all macroscopically detectable disease, whereas neoadjuvant chemotherapy followed by interval debuking surgery. After surgery, the patients can receive either carboplatin plus paclitaxel followed by endocrine therapy or endocrine therapy alone. Molecularly targeted agents, and especially MEK inhibitors and Cyclin-dependent kinase (CDK) inhibitors, are currently under evaluation. Additional research on the genomics of LGSOC and clinical trials on the combination of MEK inhibitors with hormonal agents, other molecularly targeted agents or metformin, are strongly warranted to improve the prognosis of patients with this malignancy.

## 1. Introduction

Low-grade serous ovarian carcinoma (LGSOC) is a distinct pathologic and clinical entity, accounting for 1.02–3.64% of all ovarian carcinomas and 4.66% of serous ovarian carcinomas, and characterized by younger age at presentation, less aggressive biological behavior, lower sensitivity to chemotherapy and longer overall survival (OS) compared with high-grade serous ovarian carcinoma (HGSOC) [1,2,3,4,5,6,7,8]. Patients have a median age ranging from 41.7 to 55.5 years and most of them have advanced disease at presentation. Histologically, LGSOC is characterized by a monotonous proliferation of cuboidal, low columnar cells, mild to moderate atypia without nuclear pleomorphism, mitotic index up to 12 mitoses per 10 high power fields (HPF), and destructive invasion [1,8,9]. This last histological feature can show different architectural patterns; i.e., micropapillary and/or complex papillary; compact cell nests; inverted macropapillae; cribriform; glandular and/or cystic; solid sheets with slit-like spaces and single cells [9]. Most LGSOCs are associated with a serous borderline ovarian tumor (SBOT) with micropapillary/cribriform pattern, and their invasive component usually contains a micropapillary pattern [7].

Estrogen receptors (ERs) and progesterone receptors (PRs) are expressed more frequently in LGSOC than in HGSOC [10,11,12,13,14]. The different positivity rates of steroid receptors reported in the literature are mainly due to the different immunohistochemical analytic platforms and the cut-offs used to define positivity (Table 1).

LGSOC usually develops from SBOT, and only occasionally as a de novo malignancy from the ovary or peritoneum [8,15,16,17,18]. LGSOC has been hypothesized to arise from a serous cystadenoma or adenofibroma which progresses to SBOT, to non-invasive micropapillary serous borderline tumor and then to invasive micropapillary serous borderline tumor in a slow stepwise fashion [19]. 

Gene expression profiles of LGSOCs and SBOTs are very similar [20,21] but less than 7% of SBOTs progress to LGSOCs after a lag-time ranging from 7 to 288 months [22], with the micropapillary subtype of SBOT having the highest risk of transformation [23]. 

LGSOC often harbors activating mutations of genes involved in the mitogen activated protein kinase (MAPK) pathway, such as KRAS, BRAF, ERBB2, and NRAS, but sometimes it presents driver mutations of PIK3CA, FFAR1, USP9X and EIF1AX linked to the AKT-mTOR pathway [24,25,26,27]. For instance, Grisham et al. [26] reported that RAS and BRAF mutations occurred in 38% and 31% of samples, respectively, and that these mutations were mutually exclusive. BRAF mutations appear to be more frequent in early than in advanced disease, thus suggesting that aggressive LGSOCs can arise from SBOTs without BRAF mutation [28,29]. Compared with HGSOC, LGSOCs have overexpression of insulin-like growth factor 1 [IGF-1] [30] and much lower frequency of p53 mutations [31]. BRCA mutations are extremely rare in LGSOC [32] However, a BRCA test is recommended at the time of initial diagnosis in all patients with non-mucinous and non-borderline ovarian carcinoma, fallopian tube carcinoma and primary peritoneal carcinoma [33]. The lack of MRE11, RAD50 and NBS1 protein detection, suggestive of mismatch repair (MMR) deficiency, has been recently detected in LGSOCs [34].

## 2. Diagnosis and Preoperative Work-up

The symptoms and signs of patients with LGSOC are similar to those with ovarian cancer in general. Ultrasound, which is commonly used in the initial assessment of an adnexal mass, can be of some utility in predicting LGSOC or SBOT [35,36,37]. However, there are no significant studies in the literature that have extensively evaluated the role of ultrasound in differentiating LGSOC from HGSOC. Noninvasive LGSOCs usually appear as multilocular-solid cysts with papillary projections, invasive LGSOCs present as multilocular cysts with solid component or papillary projections and extensive calcification, whereas HGSOCs look like as non-papillary solid masses with scattered areas of cystic change, hemorrhage or necrosis. Real-time ultrasound elastography can offer some additional information [38]. High-grade lesions are less stiff than low-grade lesions due to rapidly developing necrosis, whereas low-grade lesions grow slowly and their solid areas are stiffer and less elastic. 

Magnetic resonance imaging (MRI) and computed tomography (CT) can give additional information [35,37,39,40,41]. LGSOC appears as a solid or mixed solid cystic or cystic mass with mural nodularity, often with calcification. Calcified peritoneal lesions can sometimes be seen. The differential diagnosis with other pelvic masses that can calcify includes exophytic uterine fibroids and Brenner tumors, fibromas and mature cystic teratomas of the ovary. 

18F-fluorodeoxyglucose (18F-FDG) positron emission tomography (PET)/CT has a limited role in the preoperative diagnostic assessment of LGSOC, since there is no correlation between the degree of tumor maximum standard uptake value (SUVmax) of 18F-FDG and the histologic grade of ovarian tumors [42]. 

The preoperative assessment of the women is the same as those with any suspicious ovarian mass and should include the CA 125 assay and chest, abdomen and pelvic CT scans to assess tumor spread [8]. Serum CA 125 is usually above the cut-off value, but its levels are lower than those measured in patients with HGSOC.

## 3. Therapy

### 3.1. Surgery

Primary surgery is the cornestone of treatment of LGSOC [2,8,13,43,44,45,46,47,48]. Patients with disease apparently confined to the gonad(s) should undergo bilateral salpingo-oophorectomy, total hysterectomy and comprehensive peritoneal and retroperitoneal staging, although fertility-sparing surgery including unilateral salpingo-oophorectomy, surgical staging and endometrial biopsy could be taken into consideration in young women with stage IA-IC_1_ disease who strongly desire to preserve their childbearing potential. Patients with advanced disease should undergo primary debulking surgery (PDS) with the aim of removing all macroscopically detectable disease.

However, there is a small percentage of patients who may not be candidates for PDS due to the extent of disease. Neoadjuvant chemotherapy followed by interval debulking surgery (IDS) should be reserved to these patients, who usually experience a poorer prognosis

A subset analysis of GOG 182 trial, comparing carboplatin [CBCDA] + paclitaxel [PTX] alone versus combination with triplet or sequential doublet regimens, assessed 189 patients with FIGO stage III-IV LGSOC [43]. The women, who had no gross residual disease (RD) after PDS, experienced better progression-free survival (PFS) (median, 33.2 months) and OS (median, 96.9 months) compared with those with RD 0.1–1 cm (14.7 months and 44.5 months, respectively) and those with RD >1 cm (14.1 months and 42.0 months, respectively) (PFS, *p* < 0.001; OS, *p* < 0.001).

In a retrospective Italian investigation, PDS was performed in 68 of 72 patients (94.4%) with FIGO stage IIIb-IV LGSOC and achieved RD = 0 in 44 patients (64.7%), RD ≤ 1 cm in 49 (72.1%), and RD > 1 cm in 19 (27.9%) [48]. IDS was then carried out in 15 of the 19 women with RD > 1 cm after PDS and in 4 patients who did not attempt PDS. Overall, 12 of the 19 (63.1%) patients who underwent IDS had macroscopic RD after operation. Multivariate analysis showed that non-optimal cytoreduction was an independent poor prognostic factor for the risk of recurrence (hazard ratio [HR] = 2.79; 95% confidence interval [CI] = 1.16–6.70; *p* = 0.021) and the performance of IDS instead of PDS was an independent poor prognostic variable for the risk of death (HR = 2.95; 95% CI = 1.12–7.74; *p* = 0.027).

Neoadjuvant platinum ± taxane-based chemotherapy obtained a complete radiological response in one (4.2%) and stable disease in 21 (87.5%) of the 24 patients with LGSOC [49]. Nineteen patients underwent IDS, which was optimal in 12 cases (63.2%), suboptimal in 6 (31.6%) and unknown in one case (5.2%). For the entire cohort, median PFS was 21.4 months and median OS was 56.1 months.

Secondary cytoreductive surgery (SCS) may have a role in the management of recurrent LGSOC [50,51]. 

A retrospective study of Johns Hopkins Medical Institutions assessed 26 patients with ovarian micropapillary serous carcinoma, of which 21 underwent SCS and 15 (71.4%) achieved an optimal cytoreduction to RD ≤ 1cm [50]. Patients undergoing optimal SCS had longer median survival after recurrence (61.2 months) compared with either patients undergoing suboptimal SCS (25.5 months, *p* < 0.02) or not surgically-treated patients (29.9 months, *p* < 0.01), and the difference retained statistical significance on multivariate analysis (*p* = 0.01). 

A retrospective investigation of MD Anderson Cancer Center evaluated 41 patients who underwent SCS for recurrent LGSOC after a median time of 33.2 months from PDS [51]. Median survival after SCS was longer for the nine patients with no macroscopic RD than for the 32 patients with gross RD (93.6 months versus 45.8 months, *p* = 0.04). Women who were initially treated with SCS at the time of recurrence showed a trend towards a longer median survival than those who underwent chemotherapy followed by SCS (83.3 versus 33.2 months, *p* = 0.09). 

### 3.2. Chemotherapy and Endocrine Therapy

LGSOC is an indolent neoplasia less responsive to chemotherapy both in first-line and in the recurrent setting compared with HGSOC [5,6,8,46,50,52]. An in vitro study showed that LGSOC was quite resistant to PTX, CBDCA, cyclophosphamide, gemcitabine and cisplatin and less likely to be resistant to etoposide, doxorubicin and topotecan [53]. 

The metadata base of four AGO-OVAR phase III trials, including patients with FIGO stage IIIb-IV ovarian carcinoma who underwent PDS followed by platinum /PTX- based regimens, identified 145 patients with LGSOC, of which 39 (24.1%) had RD > 1 cm and were evaluable for response to chemotherapy [46]. An objective response was obtained in 10 patients (23.1%) and this response rate was significantly lower compared to 90.1% response rate in the 80 matched control women with HGSOC with RD > 1 cm (*p* < 0.001).

Twelve patients with measurable recurrent LGSOC received platinum-based salvage chemotherapy, with the most common regimen being a platinum agent combined with PTX [50]. Two patients achieved a complete response and one patient achieved a partial response, for an overall response rate of 25.0%, and three patients (25.0%) had stable disease. The analysis of the departmental databases of MD Anderson Cancer Center reported one complete response and three partial responses following 108 different chemotherapy regimens to 58 patients with recurrent LGSOC, for on overall response rate of 3.7%, ranging from 2.1% for platinum-resistant to 4.9% for platinum-sensitive patients [51]. The stable disease rate was 60.2% and the median time to progression was 29.0 weeks. 

Hormonal therapy may provide clinical benefit in women with LGSOC [11,13,54,55,56]. Escobar et al. [11] found ER+/PR+ in 21.8%, ER + /PR- in 17.4%, ER- /PR+ in 13.0% and ER- /PR- in 47.8% of 27 LGSOCs and assumed that only the subset of tumors with double steroid receptor expression could benefit from endocrine treatment.

Gershenson et al. [55] examined 203 patients with FIGO stage II-IV LGSOC who underwent PDS and platinum-based chemotherapy, followed by hormone therapy in 70 cases and observation alone in 133 cases. Hormone therapy consisted of letrozole in 38 cases (54.3%), anastrozole in 2 (2.9%), tamoxifen in 20 (28.6%), leuprolide acetate as single agent or in combination in 9 (12.9%), and medroxyprogesterone acetate in one (1.4%) case. Patients who had maintenance endocrine therapy had better PFS (median, 64.9 versus 26.4 months, *p* < 0.001) than those who had not, whereas OS was similar in the two groups (115.7 versus 102.7 months, *p* = 0.42). 

A retrospective study of Johns Hopkins School of Medicine, Cleveland Clinic and Memorial Sloan Kettering Cancer Center assessed 27 patients with FIGO stage II–IV LGSOC who underwent either PDS (*n* = 26) or neoadjuvant CBDCA/PTX-based chemotherapy plus IDS (*n* = 1) followed by endocrine therapy, consisting of letrozole (55.5% of cases), anastrozole (37.1%) or tamoxifen (7.4%), for a median time of 18 months [13]. Six (22.2%) patients relapsed after a median time of 21.5 months, 3-year PFS was 79.0% and 3-year OS was 92.6%. It is noteworthy that none of the three patients who had tumor tissue collected for molecular analysis at recurrence showed ESR1 mutations which have been associated with resistance to aromatase inhibitors in ER+ metastatic breast cancer [57].

A randomized phase III trial is currently comparing CBDCA/PTX for six cycles followed by maintenance letrozole versus letrozole monotherapy after PDS in women with stage II-IV LGSOC (NRG-GY-019).

The M.D. Anderson Cancer Center analyzed 64 patients who received 89 different hormone regimens for recurrent LGSOC [54]. There were six complete responses and two partial responses, for an overall response rate of 9.0% in the entire cohort, 2.7% in platinum-resistant patients and 13.5% in platinum-sensitive patients. The objective responses were achieved in six of the 33 cases (18.2%) treated with letrozole, one of the 21 (4.8%) treated with anastrozole, one of the 17 (5.9%) treated with tamoxifen, none of the 14 treated with leuprolide alone or combined with other agents, and none of the four treated with fulvestrant, megestrol acetate or raloxifene. Median to progression was 7.4 months in the entire cohort, 8.9 months in patients with ER+/PR+ disease and 6.2 months in those with ER+ /PR- disease. 

Anastrozole obtained a partial response in 5 (13.9%), 3-month clinical benefit (partial response + stable disease) in 23 (63.9%) and a 6-month clinical benefit in 21 (60.8%) of the 36 post-menopausal women with ER+ and/or PR+ recurrent/metastatic LGSOCs and SBOTs [56]. The median PFS was 11.1 months.

An ongoing basket phase II study is assessing the oral progesterone antagonist onapristone ER(Apristor) in patients with PR+ recurrent LGSOC, ovarian granulosa cell tumor or endometrioid endometrial carcinoma (NCT03909152).

### 3.3. Molecularly Targeted Agents

Different molecularly targeted agents have been tested in LGSOC (Table 2) 

#### 3.3.1. Bevacizumab

Single-agent bevacizumab achieved a dramatic response lasting from 15 to 22 months in three patients with recurrent disease [58]. An extended (>7 years) activity of bevacizumab + metronomic cyclophosphamide was observed in one patient with platinum-resistant LGSOC [59].

A retrospective investigation of Memorial Sloan Kettering Cancer Center identified 17 patients with recurrent LGSOC treated with bevacizumab as single agent (*n* = 2) or in combination with different chemotherapeutics (*n* = 15) [60]. A partial response was obtained in six (40.0%) and a stable disease lasting ≥ 3 months was observed in five (33.3%) of the evaluable 15 patients. The 5-year OS was 61.8%. Weekly PTX was the most common chemotherapy administered in combination with bevacizumab and it accounted for five of the six partial responses. 

Rose et al. [61] treated 12 patients with LGSOC with bevacizumab as a single agent (*n* = 11) or in a combination regimen (*n* = 1). Only one (8.3%) patient achieved a partial response, but three (25.0%) patients had stable disease that lasted for 123+, 48, and 15+ months after progression-free intervals on prior chemotherapy of 2.5, 4, and 7 months, respectively. Therefore, some patients had secondary PFS durations longer than their prior PFS. 

Dalton et al. [62] analyzed 40 patients with recurrent LGSOC who received a bevacizumab -containing regimen. Five of these were treated with bevacizumab on two separate occasions. Three and 16 patients obtained a complete response and a partial response, respectively, for an overall response rate of 47.5%, whereas 12 patients (30.0%) had stable disease. The median PFS was 10.2 months and median OS was 34.6 months. Bowel perforation and entero-cutaneous fistula occurred in two patients and one patient, respectively.

#### 3.3.2. MEK Inhibitors

The high prevalence of MAPK pathway alterations in LGSOC and the limited activity of chemotherapy has stimulated the evaluation of selective MEK inhibitors in patients with this malignancy [25,26,27,28,63,64,65].

Trametinib obtained a sustained response in a 56- year old woman with recurrent highly pretreated LGSOC harboring NRAS mutation [64].

Selumetinib (50 mg twice daily orally) was given to 52 patients with recurrent LGSOC [63]. A complete response and a partial response were achieved in one and seven patients, respectively, for an overall response rate of 15.4%, and a stable disease was observed in 34 patients (65.4%). The response to selumetinib was not related to RAS/RAF mutational status. Median time to response was 4.8 months, median duration of response was 10.5 months, and median PFS was 11 months. The most frequent, but manageable, adverse events were grade 3 gastrointestinal toxicity (25.0%) and grade 3 dermatologic toxicity (17.3%) 

A phase II study randomly allocated 65 patients with previously treated, recurrent LGSOC or SBOT with measurable disease to receive either the combination of pimasertib (60 mg daily) + voxtalisib (70 mg daily) or pimasertib alone (60 mg twice daily) [65]. There was no significant difference in partial response rate (12.1% versus 9.4%), stable disease rate (36.4% versus 50.0%), and 6-month PFS (70.8% versus 63.5%) between pimasertib arm and combination arm. Eighteen (56.3%) patients of the combination arm and 19 (57.6%) of the pimasertib arm discontinued the trial, and therefore the study was early stopped because of low response rate and high discontinuation rate. 

The MILO phase III study, which randomized patients with recurrent or persistent LGSOC to receive either binimetinib or physician’s choice chemotherapy (liposomal doxorubicin, PTX, topotecan), was prematurely closed due to futility (NCT01849874). A phase II/III trial has randomized patients with recurrent or progressive LGSOC to undergo either trametinib or standard treatment (with either letrozole, tamoxifen, PTX, pegylated liposomal doxorubicin, or topotecan). The accrual has been completed but the definitive results are not yet available (NCT02101788).

Based on recent studies, it has been hypothesized that the main oncogenic signaling pathway is PIK3CA/AKT in Asian women (mutation rate = 60%) and KRAS/BRAF/ERK (mutation rate = 16–54%) in Western women, which suggests that the efficacy of molecularly targeted agents could be race-dependent [24,66,67]. 

Metformin can exert antitumor effects mainly through AMP-activated protein kinase [AMPK] activation and PI3K-mTOR inhibition [68,69]. Moreover, metformin decreases the production of insulin, IGF-1 and vascular endothelial growth factor, and therefore it exerts anti-mitotic and anti-angiogenetic effects. In vitro studies showed that metformin inhibited all LGSOC cell lines, whereas trametinib significantly inhibited the growth of RAS-mutated LGSOC cell lines (VOA1312 and VOA1056) but not of VOA5646 cells without RAS mutation [70]. Therefore, metformin alone or in combination with MEK inhibitors could be helpful in the management of LGSOC.

#### 3.3.3. Cyclin-Dependent Kinases Inhibitors

Ribociclib has been approved by the Food and Drug Administration and the European Commission as initial endocrine-based therapy for postmenopausal women with hormone receptor positive, human epidermal growth factor receptor 2 (HER2)-negative advanced breast cancer in combination with an aromatase inhibitor following a randomized phase III trial [71]. A phase II study is currently assessing the combination of letrozole (2.5 mg oral daily) + ribociclib (600 mg oral daily for 3 weeks then one week off) in women with recurrent LGSOC (NCT03673124). Primary outcome measure is response rate, secondary outcome measures are clinical benefit rate, toxicity, PFS and OS, and other outcome measures include ER and PR expression, mutation analysis of genomic signatures, and Ki-67 expression. These biological variables will be correlated with response and clinical benefit.

A pilot phase II study of neoadjuvant fulvestrant (500 mg im. on days 1 and 15 of cycle 1 and day 1 of cycles 2–4) plus abemaciclib (150 mg oral on days 1–28 of each cycle) every 4 weeks is ongoing in patients with stage III-IV LGSOC (NCT03531645). The primary endpoint is the clinical benefit rate after four cycles of treatment. Besides being the most potent CDK4/6 inhibitor, abemaciclib displays a wider selectivity towards other CDKs and kinases, and it functions through additional mechanisms besides inducing G1 cell cycle arrest [72].

## 4. Prognosis

Patients with LGSOC experience a significantly better prognosis than those with HGSOC [1,2,3,7,8].

An investigation of the National Cancer Institute’s SEER program showed that mean OS was 99 months for LGSOC versus 57 months for HGSOC [3]. Okoye et al. [7] found that patients with LGSOC had better 5-year OS than those with HGSOC (62.3% versus 43.9%), but this advantage decreased over time and the corresponding 10- year OS rates were similar (21.2% versus 22.7%) 

RD is a strong prognostic variable in advanced LGSOC [14,43,44,46]. Among the women with LGSOC identified from AGO-metadatabase, 5-year OS was 85% for the patients who underwent complete cytoreduction versus 32% for those with RD > 1 cm (*p* < 0.001) [46]. In a Canadian study including 55 patients with FIGO stage III-IV LGSOC, the risk of recurrence and death was 4.31-fold higher (95% CI = 1.30–14.26) and 5.35-fold higher (95% CI = 1.41–20.3), respectively, in patients with RD > 1cm compared with those with RD = 0 [14].

In the ancillary analysis of the GOG 182 trial on patients with LGSOC, a pretreatment CA 125 assay had no prognostic relevance [41]. However, patients with CA 125 levels in the normal range after cycle 1, 2 or 3 were 60%-64% less likely to experience progression as compared to those who never normalized or normalized after four cycles.

The analysis of 287 patients with stage II-IV LGSOC who underwent PDS followed by platinum-based chemotherapy at MD Anderson Cancer Center found that women older than 35 years at diagnosis had longer PFS (median = 31.2 versus 17.8 months, HR = 0.55, 95% CI = 0.38–0.81, *p* = 0.002) and longer OS (median = 102.9 versus 72.8 months, HR = 0.55, 95% CI = 0.39–0.79, *p* = 0.001) compared to younger women [6].This study failed to detect a negative prognostic impact of smoking and body mass index (BMI) ≥ 35 mg/kg. 

Ahn et al. [9] assessed the clinico-pathologic features of 52 women with LGSOC diagnosed at three US Departments of Pathology. Five-year PFS was 47% and 5-year OS was 82%. All the patients who died had tumors showing invasion with predominant patterns of cribriform glands, micropapillae and/or complex papillae, or compact cell nests. 

A high composite ER Allred score, based on both proportionate and immunostaining intensity score for ER expression, was an independent good prognostic variable for OS in a Canadian study on patients with advanced LGSOC [14]. Conversely, low levels of PR were associated with a more aggressive clinical course.

Whereas in advanced colorectal and lung cancer KRAS and BRAF mutations are predictive of worse clinical outcome [73,74], Gershenson et al. [75] reported a better OS in the 21 patients whose LGSOCs contained a KRAS or BRAF mutation compared to the 58 patients whose LGSOCs had no KRAS or BRAF mutations (median = 106.7 versus 66.8 months, HR = 0.49, 95% CI = 0.26–0.95; *p* = 0.03). 

## 5. Conclusions

LGSOC has a less aggressive behavior and a better clinical outcome compared with HGSOC. Surgery is the keystone of treatment, and considering that this malignancy is not very chemo-sensitive, an attempt of primary maximal cytoreduction is strongly warranted [40]. Women with stage IA—IB disease should undergo observation alone after comprehensive staging surgery, whereas observation, chemotherapy or endocrine therapy are all possible options for those with stage IC-IIA disease [13,55,76]. Patients with stage IIb-IV disease can receive either chemotherapy with CBDCA + PTX for six cycles followed by endocrine therapy, most commonly consisting of aromatase inhibitors, or endocrine therapy alone until disease progression or unacceptable toxicity. While some authors [11] have assumed that only patients with both ER and PR expression can benefit from hormonal treatment, others [14,77] have suggested that additional translational research is warranted to identify the molecular predictors of response to endocrine therapy. We must take into consideration that the biological effects of estrogens are mediated by two distinct receptors: ERα, which exerts proliferative and pro-tumorigenic effects, and ERβ, which displays anti-proliferative, pro-apoptotic and anti-metastatic activity [78]. Therefore, the assay of relative levels of ERα and ERβ isoforms should be included in future clinical studies on the endocrine treatment of LGSOC [77]. 

There is no agreement in the literature about the type and timing of examinations to perform for the follow-up of patients after primary treatment for ovarian cancer in general [79]. A minimalist protocol consists of periodical history, physical examinations and CA125 assay, while an intensive approach also includes planned diagnostic imaging procedures in asymptomatic patients. The surveillance programs for patients with LGSOC are the same used for those with ovarian carcinoma in general [37]. However, a post-therapy increase in peritoneal implant calcification on a CT scan is not suggestive of a response to treatment, differently from other malignancies such as lymphoma, colorectal cancer and glioblastoma [80].

SCS, chemotherapy and endocrine therapy are used also in patients with recurrent disease. Molecularly targeted agents, and especially MEK inhibitors and CDK inhibitors, are currently under evaluation, especially in this clinical setting. Additional research on the genomics of LGSOC, to better define the activating gene mutations involved in the carcinogenesis, and clinical trials on the combination of MEK inhibitors with hormonal agents, other molecularly targeted agents (BRAF inhibitors, PI3K/mTOR inhibitors, IGF-1R–targeted therapy) or metformin, are strongly warranted to improve the prognosis of patients with this malignancy.

## Figures and Tables

**Table 1:** Estrogen receptor (ER) and progesterone receptor (PR) expression in low-grade serous ovarian carcinoma.

Authors [Ref]	Patients (*n*)	ER	PR
Wong [10]	47	58%	43%
Escobar [11]	26	96%	44%
Sieh [12]	110	88%	57%
Fader [13]	25	96%	32%
Fernandez Laudano [14]	55	96%	67%

**Table 2:** Molecularly targeted agents tested in low-grade serous ovarian carcinoma.

Antiangiogenic Agent	Bevacizumab
MEK inhibitors	Trametinib Selumetinib Pimasertib Binimetinib
PI3K inhibitor	Voxtalisib
Cyclin-dependent kinase (CDK) 4/6 inhibitors	Ribociclib Abemaciclib
